# Supplementary material for: Mutational sources of trans-regulatory variation affecting gene expression in Saccharomyces cerevisiae
Source: eLife. 2021 Aug 31;10:e67806. doi: 10.7554/eLife.67806 (PMC8456550; doi:10.7554/eLife.67806)
Supplement: Transparent reporting form [file elife-67806-transrepform.docx]

***eLife’s* transparent reporting form**

We encourage authors to provide detailed information *within their submission* to facilitate the interpretation and replication of experiments. Authors can upload supporting documentation to indicate the use of appropriate reporting guidelines for health-related research (see [EQUATOR Network](http://www.equator-network.org/%20)), life science research (see the [BioSharing Information Resource](https://biosharing.org/)), or the [ARRIVE guidelines](http://www.plosbiology.org/article/info:doi/10.1371/journal.pbio.1000412) for reporting work involving animal research. Where applicable, authors should refer to any relevant reporting standards documents in this form.

If you have any questions, please consult our Journal Policies and/or contact us: [editorial@elifesciences.org](mailto:editorial@elifesciences.org).

**Sample-size estimation**

- You should state whether an appropriate sample size was computed when the study was being designed
- You should state the statistical method of sample size computation and any required assumptions
- If no explicit power analysis was used, you should describe how you decided what sample (replicate) size (number) to use

Please outline where this information can be found within the submission (e.g., sections or figure legends), or explain why this information doesn’t apply to your submission:

The total number of mutant strains included in the mapping experiment (82 strains) was chosen to retrieve a number of mutations sufficiently large for meaningful comparisons of the properties of *trans*-regulatory and non-regulatory mutations. This number was limited by the time and the cost necessary for mapping mutations in each mutant strain.

As mentioned in Results and Methods, we previously published a detailed power analysis (in Duveau et al., 2014) to determine critical parameters of the mapping experiments such as population size, strength of selection and sequencing depth.

For expression assays and fitness assays, sample sizes were chosen based on our previous studies using the same fluorescent reporter and the same flow cytometer (Gruber et al., 2012; Metzger et al., 2015; Metzger et al., 2016; Duveau et al., 2017; Duveau et al., 2018).

**Replicates**

- You should report how often each experiment was performed
- You should include a definition of biological versus technical replication
- The data obtained should be provided and sufficient information should be provided to indicate the number of independent biological and/or technical replicates
- If you encountered any outliers, you should describe how these were handled
- Criteria for exclusion/inclusion of data should be clearly stated
- High-throughput sequence data should be uploaded before submission, with a private link for reviewers provided (these are available from both GEO and ArrayExpress)

Please outline where this information can be found within the submission (e.g., sections or figure legends), or explain why this information doesn’t apply to your submission:

Numbers of replicate populations for each genotype in expression and fitness assays are indicated in figure legends and in Materials and Methods. These replicates represent independent cultures of a given clonal strain. Outliers were removed as described in Materials and Methods.

High-throughput sequencing data are publicly available from NCBI SRA (<https://www.ncbi.nlm.nih.gov/sra>) under BioProject number PRJNA706682. Flow cytometry data are publicly available on the Flow Repository ([https://flowrepository.org/](https://flowrepository.org/))) under experiments ID FR-FCM-Z3WV, FR-FCM-Z3JY, FR-FCM-Z3J2, FR-FCM-Z3J3 and FR-FCM-Z3J5. This information is provided in a section of the manuscript entitled “Data archiving”.

**Statistical reporting**

- Statistical analysis methods should be described and justified
- Raw data should be presented in figures whenever informative to do so (typically when N per group is less than 10)
- For each experiment, you should identify the statistical tests used, exact values of N, definitions of center, methods of multiple test correction, and dispersion and precision measures (e.g., mean, median, SD, SEM, confidence intervals; and, for the major substantive results, a measure of effect size (e.g., Pearson's r, Cohen's d)
- Report exact p-values wherever possible alongside the summary statistics and 95% confidence intervals. These should be reported for all key questions and not only when the p-value is less than 0.05.

Please outline where this information can be found within the submission (e.g., sections or figure legends), or explain why this information doesn’t apply to your submission:

Statistical analyses are explained in detail in Materials and Methods. R scripts used to perform statistical analyses are available in Source Code 1-3. P-values supporting the main results are indicated in the main text. All P-values are reported in datasets included in Supplementary Files.

(For large datasets, or papers with a very large number of statistical tests, you may upload a single table file with tests, Ns, etc., with reference to sections in the manuscript.)

**Group allocation**

- Indicate how samples were allocated into experimental groups (in the case of clinical studies, please specify allocation to treatment method); if randomization was used, please also state if restricted randomization was applied
- Indicate if masking was used during group allocation, data collection and/or data analysis

Please outline where this information can be found within the submission (e.g., sections or figure legends), or explain why this information doesn’t apply to your submission:

For fitness and expression assays, samples were randomized in 96-well plates as indicated in Materials and Methods. Plate positions of all genotypes are indicated in Supplementary File 12/Flow.Template.

**Additional data files (“source data”)**

- We encourage you to upload relevant additional data files, such as numerical data that are represented as a graph in a figure, or as a summary table
- Where provided, these should be in the most useful format, and they can be uploaded as “Source data” files linked to a main figure or table
- Include model definition files including the full list of parameters used
- Include code used for data analysis (e.g., R, MatLab)
- Avoid stating that data files are “available upon request”

Please indicate the figures or tables for which source data files have been provided:

Source data are provided for all figures displaying quantitative data and can be found in SourceData.tar.bz2 (compressed folder containing 34 tab-delimited text files). This includes Figure 1B-E, Figure 2E-G, Figure 3A,B,E, Figure 5C-G, Figure 6, Figure 7, Figure 2 – figure supplements 1-5, Figure 3 – figure supplements 1-4 and Figure 7 – figure supplement 1.
